# Mucinous adenocarcinoma of the bladder associated with long term suprapubic tube: a case report

**DOI:** 10.1186/s12894-015-0112-8

**Published:** 2015-12-03

**Authors:** Tyler M. Bauman, Theodora A. Potretzke, Aaron M. Potretzke, Cary L. Siegel, Steven B. Brandes

**Affiliations:** Division of Urologic Surgery, Washington University School of Medicine, St. Louis, MO USA; Department of Radiology, Washington University School of Medicine, St. Louis, MO USA

**Keywords:** Adenocarcinoma, Suprapubic tube, Catheter, Bladder

## Abstract

**Background:**

Chronic indwelling catheters may induce histologic changes within the bladder, and these changes are sometimes pre-malignant. There are many documented cases of squamous cell carcinoma associated with indwelling catheters, but only three cases of catheter-associated adenocarcinoma have been reported. In this case report, we present radiographic findings of a case of mucinous adenocarcinoma of the bladder and suprapubic (SP) tract in a quadriplegic patient.

**Case presentation:**

A 71-year-old male with a history of spinal cord injury presented with hematuria and SP discharge after SP catheterization for 51 years. CT urography was performed and revealed an irregular, infiltrative, and heterogeneous mass arising from the anterior bladder at the level of the suprapubic catheter and extending along the SP tube tract. Cystoscopy and biopsy revealed an adenocarcinoma of the anterior bladder and stoma with extensive associated mucin production and a background of acute and chronic inflammation. Surgical therapy included cystoprostatectomy, abdominal wall resection, ileal conduit creation, and abdominal wall reconstruction. The final diagnosis was a high-grade, T2a/N0/M0 (Stage II) mucinous adenocarcinoma of the bladder. There has been no evidence of tumor recurrence over the previous 5 years.

**Conclusion:**

Few cases of adenocarcinoma associated with long term indwelling catheter have been reported in the literature, and due to the rarity of this disease process, the prognosis with surgical therapy is not well known. The patient described herein has been free of recurrence for the previous five years, suggesting that surgery is a viable management option for these patients.

## Background

Quadriplegia due to spinal cord injury (SCI) compromises normal urinary function. Suprapubic (SP) tube placement is favored in quadriplegic patients who are unable to perform clean intermittent catheterization, have acquired complications from urethral catheterization, or require continuous bladder drainage [1, 2]. However, chronic indwelling catheters may induce inflammatory and proliferative histologic changes in the bladder, which in some cases are pre-malignant [1]. Although cases of squamous cell carcinoma (SCC) associated with intermittent catheterization or long-term SP tube placement are found throughout the literature [2, 3], only three previous cases of catheter-associated adenocarcinoma have been reported [1, 4]. Herein, we report the case and present the radiographic findings of a mucinous adenocarcinoma of the bladder and SP tract associated with long-term indwelling SP catheter in a patient with SCI.

## Case presentation

A 71-year-old C6 quadriplegic male with a chronic indwelling SP catheter presented with hematuria and discharge from the SP tube. The patient suffered a traumatic spinal cord injury at age 19 and an indwelling SP catheter has been present for 51 years, changed monthly. Past medical history was significant for urinary tract infections (UTIs) and nephrolithiasis. Patient endorsed a 25-year history of smoking.

**Fig. 1:**
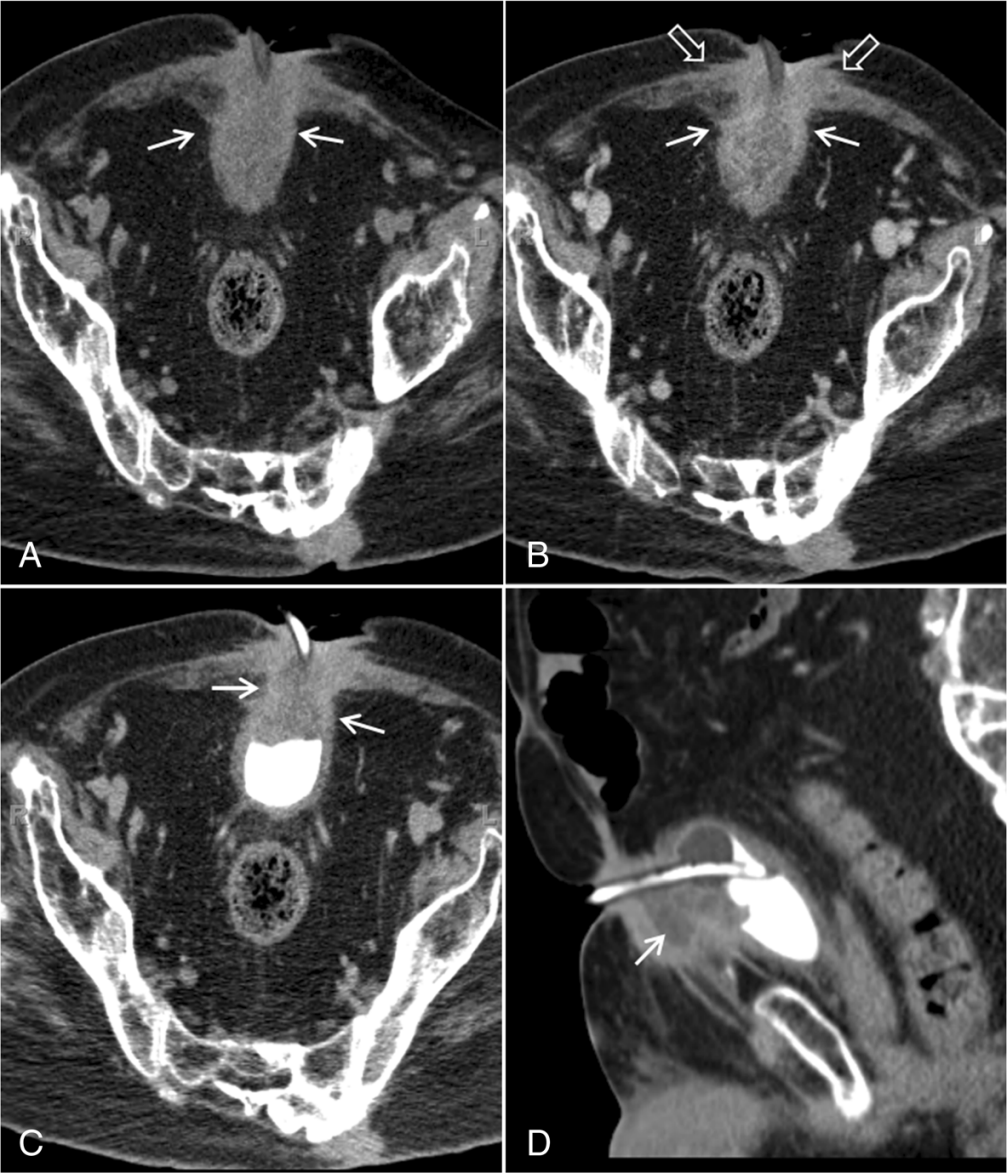
Pre- and post-contrast axial images from CT urography (**a** and **b**) as well as axial and sagittal images acquired during the delayed excretory phase (**c** and **d**) demonstrate an irregular enhancing soft tissue mass arising along the anterior bladder at the level of the suprapubic catheter and extending along the catheter tract to the skin surface (arrows in **a**–**c**). There is infiltration of the anterior abdominal wall (open arrows in **b**). A low density component of the mass (arrow in **d**) likely represents intralesional mucin

Computed tomography (CT) urography was performed and revealed an irregular, infiltrative, and heterogeneous mass arising from the anterior bladder at the level of the suprapubic catheter and extending along the SP tube tract (Fig. 1). The mass included peripheral areas of infiltrative enhancing soft tissue extending from the bladder, along the tract, and involving the anterior abdominal wall as well as fluid density components. There was no associated calcification or lymphadenopathy. There were no findings of a urachal remnant, and the mass did not extend toward the umbilicus. Cystoscopy and subsequent biopsy were performed, revealing adenocarcinoma of the anterior bladder and stoma with extensive associated mucin production and a background of acute and chronic inflammation. Patient was referred for definitive surgical management.

Extirpative therapy included cystoprostatectomy, abdominal wall resection, ileal conduit creation, and abdominal wall reconstruction. The surgical defect at the level of the skin was approximately 8 cm in diameter, and there was a 3 cm margin circumferentially around the suprapubic tube tract at the skin level. The umbilicus was superior to the involved abdominal wall, and the suspicion of a primary urachal adenocarcinoma was low based on the clinical presentation and history. As such, the umbilicus was not resected as part of the surgical specimen. The differential diagnosis included urachal carcinoma, but these cancers typically arise in the dome of the bladder and no urachal remnant was identified on imaging or at surgery [5]. Pathologic examination of the bladder revealed an invasive, moderately differentiated 2.7 cm adenocarcinoma with mucinous features arising in a background of extensive intestinal metaplasia. Invasion through the urothelial basement membrane and into the superficial lamina propria was noted. Urethral and ureteral margins were free of tumor and no lymphovascular space invasion was identified. A standard bilateral pelvic lymph node dissection was performed, including the obturator, internal iliac, and external iliac nodal packets, and no evidence of malignancy was found in any lymph nodes. The final diagnosis was a high-grade, T2a/N0/M0 (Stage II) mucinous adenocarcinoma of the bladder based upon WHO/ISUP grading and AJCC/UICC staging. Contrast-enhanced CT of the abdomen and pelvis and chest with either CT or x-ray was performed every 6 months for 2 years and every 12 months thereafter to monitor for local tumor recurrence or metastatic disease. These surveillance intervals are in accordance with the National Comprehensive Cancer Network guidelines [6]. There has been no evidence of tumor recurrence over the previous 5 years.

## Conclusions

The long-term management of the urinary function of quadriplegic patients presents unique challenges for the urologist, including elevated rates of kidney and bladder stones, renal damage, UTIs, and urinary cancers [7, 8]. Approximately 0.38 % of quadriplegic patients die of urethral, bladder, or renal cancers, compared to 0.008 % in the general public [9]. SCC of the bladder is relatively common secondary to prolonged indwelling catheterization, with a reported incidence of 10 % in patients with a catheter in place for ≥10 years [10]. This is thought to be related to chronic bladder inflammation inducing squamous metaplasia of the bladder epithelium [1]. Clean intermittent self-catheterization (CISC) reduces the incidence of bladder SCC, with reports of SCC associated with CISC found sparingly throughout the literature [3].

Cancers arising from a SP catheter tract are also a rare occurrence. As of 2014, there have been fewer than 10 reported cases of squamous cell carcinoma (SCC) of the SP tract or bladder associated with SP catheters [2]. Although less common than squamous metaplasia, glandular histologic changes including cystitis glandularis and intestinal metaplasia are also well documented in this risk group [1]. Differentiating mucinous adenocarcinomas from other carcinoma subtypes using immunohistochemistry (IHC) is difficult, as the markers CK7 and GATA3 are commonly expressed in other types of urothelial carcinomas [11]. Further, mucinous adenocarcinomas show variable expression of CK20 and CA125. Therefore, IHC was not used to help in the diagnosis for this patient, as is standard at our institution in such cases. We recommend using the clinical impression of the primary tumor site and clinical correlation with the patient’s relevant history for diagnosis. In select cases, the use of CK7 and GATA3 may prove useful in providing a definitive diagnosis. Due to the rarity of this disease process, the prognosis with surgical therapy is also not well known, but this patient has been recurrence free for >5 years. The present report marks the fourth described case of mucinous adenocarcinoma associated with a chronic indwelling catheter.

### Consent

Written informed consent was obtained from the patient for publication of this care report and any accompanying images. A copy of the written consent is available for review by the editor of this journal.

## Abbreviations

SCI: spinal cord injury
SP: suprapubic
SCC: squamous cell carcinoma
CT: computed tomography
CISC: clean intermittent self-catheterization
UTI: urinary tract infection
